# Developing Children’s Socio-Emotional Competencies Through Drama Pedagogy Training: An Experimental Study on Theory of Mind and Collaborative Behavior

**DOI:** 10.5964/ejop.v16i4.2054

**Published:** 2020-11-27

**Authors:** Macarena-Paz Celume, Thalia Goldstein, Maud Besançon, Franck Zenasni

**Affiliations:** a Laboratoire de Psychologie et d’Ergonomie Appliquées (LaPEA), Université de Paris, Paris, France; b Center for Research and Interdisciplinarity, Paris, France; c Social Skills, Imagination and Theatre Lab, Department of Psychology, George Mason University, Fairfax, VA, USA; d Laboratoire de Psychologie: Cognition, Comportement, Communication (LP3C), Université de Rennes 2, Rennes, France; University of Wroclaw, Wroclaw, Poland

**Keywords:** socio-emotional competencies, theory of mind, collaborative behavior, drama pedagogy, children

## Abstract

Drama pedagogy training (DPT) is a drama-based-pedagogy focused on socio-emotional-learning (SEL) development, over academic or artistic. This study aims to see if DPT promotes theory of mind (ToM) and collaborative behavior in 126 French children aged 9-10 years old, randomly assigned to an experimental group (DPT), either a control group for 6 weeks. Post-tests showed large effects of training on ToM, F(1, 124) = 24.36, p < .001, η² =.16, and collaborative behavior, F(1, 124) = 29.8, p < .001, η² = .19. T-test showed significant differences on ToM (t = -4.94, p < .001) and collaborative behavior (t = -5.46, p < .001), higher for DPT. Effects of type of school and grade are discussed. Results confirm the hypotheses.

Drama pedagogy training (DPT; Celume, Besançon, & Zenasni, 2019a) can be defined as any drama-based training or workshop, created through drama activities and techniques (such as pretend play, improvisation, role-play, etc.) that follows the characteristics of the liberal progressivism tendency from drama pedagogy (García-Huidobro, 1996). This approach focuses on the work of internal world of the participant through drama games instead of searching an academic or artistic achievement. Moreno’s (1943, 1947) socio-drama, Boal’s (1978, 1989) theatre of the oppressed and Slade’s (1967, 1998) drama education were some of the first approaches using representational games in the work with people focusing on competencies development instead of an artistic training, setting the beginnings of DPT as a methodology. Those ideas were also outlined by Karakelle (2009), Libman (2001) and Woodson (1999) who defended the idea of focusing on the process and experience of learning competencies without any intention of showing off. In current literature, this methodology turned into several different names, such as creative drama (Rosenberg, 1981), process drama, drama pedagogy (García-Huidobro, 2004), applied theatre (Holland, 2009) or even child drama, play making, child play, or educational drama. What they all have in common, is that their focus is non academic, nor artistic, even if some of these concepts describe drama practices used in education for educational objectives (Freeman, Sullivan, & Fulton, 2003). DPT could be also considered as a specific kind of training within drama based pedagogy (for a review see Lee, Patall, Cawthon, & Steingut, 2015), based on drama pedagogy theory (García-Huidobro; 2004) and which is focused only on participant’s socio-emotional and creative development, and thus, not created to enhance any academic field, or to contribute to any subject in school, neither to train participants in any artistic way. According to Celume et al. (2019a) DPT is a methodology that encompasses drama activities and techniques through the use of embodiment and narratives, under a particular psycho-pedagogical framework that works with the use of narrative and the construction of a dialogic space (Celume, Besançon, & Zenasni, 2019b). DPT can be considered as an active methodology, that works with the cognitive and affective dimensions of the participants emphasizing the importance of playfulness and positive emotions, promoting a safe and caring environment and a positive student-pedagogue relationship (Garaigordobil & Berrueco, 2011; García-Huidobro, 2004; Hammond, 2015; Rosen, 1974) that would develop social and emotional competencies and skills.

An important part of successful socio-emotional competencies programs are based on DPT elements such as role playing, improvisation, pretend play, storytelling and others. These conclusions can be consulted in recent meta-analyses (Durlak, Weissberg, Dymnicki, Taylor, & Schellinger, 2011; Lee et al., 2015) that showed strong relationships between the trainings they reviewed and the socio-emotional outcomes measured. Nevertheless, the meta-analysis conducted by Durlak et al. (2011), is identified as a socio-emotional learning programs review, and thus is not focused on drama pedagogy trainings, but on different socio-emotional school-based interventions, even though several of the successful interventions reviewed used drama pedagogy elements within their trainings. Durlak et al. (2011) showed that in general, the programs reviewed had positive effects on the socio-emotional competencies willing to develop, and that these interventions worked better on children than adolescents. They also observed that the use of a specific protocol (ref. SAFE protocol) moderated positive students’ outcomes, which corresponds with the description of the psycho-pedagogical framework of DPT (Celume et al., 2019a). In the same line, interventions that were identified as more interactive and that, for example, used several DPT elements, such as role playing, were the most successful among young students. Similarly, Garaigordobil et al. (1996), compared 6 experimental groups versus 2 control groups, in order to see the effect of a game-based intervention (that included drama-based games) on collaborative behavior. Results showed significant differences between the experimental and the control groups, *t*(34) = 5.65, *p* < .001, meaning that an intervention of this kind was able to develop competencies in the field of reciprocal help behaviors. Regarding the development of other socio-emotional competencies such as empathy or theory of mind (ToM) through drama-based pedagogies, Goldstein and Winner (2012) conducted two studies in children and adolescents looking for relationships between drama, empathy and ToM, and finding that these skills could be enhanced through drama. Results for the elementary school children sample showed a higher impact of acting training on empathy over controls, *F*(1, 64) = 4.26, *p* = .043, *d* = 0.53, but no significant scores were found for ToM, probably due to a possible issue related to the measurement tool chosen for assessing ToM. In the same line, Celume and Zenasni (in press) conducted an observational study where they observed and analyzed DPT sessions, outlining perspective taking as un underlying mechanism that permitted the development of theory of mind and creativity in elementary school children.

Nevertheless, a review on pretend play and its effects on child development (Lillard et al., 2013), found that correlations between pretend play and social skills, and pretend play and ToM were inconsistent, although most of the studies reviewed showed “important failures to replicate” (p. 16), which could be an explanation to the lack of positive results. In a similar way, the meta-analysis of Lee et al. (2015) studied the effect of drama based pedagogies (DBP) on children from preschool to adolescence, finding that DBP had a significant impact on educational achievement outcomes but that the impact on social skills was not significant, concluding that the belief that DBP might promote prosocial behaviors was *unfounded*.

On the contrary, it was supported that DBP had a significant positive effect on other children’s social skills, like attitudes toward achievement and the so-called 21st century skills (which includes collaborative behavior). Moreover, several drama-based trainings were considered to be out of the scope and thus weren’t examined in the meta-analysis, we missed, for example, some well-known drama-based methodologies, such as the Speech Bubbles project or the Drama for Learning and Creativity (D4LC), which we believe could have influenced some of the negative results regarding DBP and its impact on social competencies. In the same line, the meta-analysis of Lee et al. (2015) explains that they had to exclude several studies from their analysis due to a lack of reporting information, demanding that researchers must better report their methods, as it is fundamental for replicability. They found that most of the studies analyzed presented important bias as they were mostly quasi-experimental studies that rarely presented matching control groups, being difficult to assure that DBP was or was not the cause of social outcomes enhancement, concluding that there is a need of better reporting the trainings and strategies used in order to be able to have consistent material to analyze and thus have consistent results.

To our knowledge, and based on these meta-analyses, except for the recent work from Goldstein and Lerner (2018) and a study on fantasy play (e.g. Thibodeau, Gilpin, Brown, & Meyer, 2016), studies that have been done in order to examine the impact of DPT methods and elements on socio-emotional competencies relies on studies a) without an active control group well matching the experimental one, b) where children volunteer to participate in the experimental group and so there’s no randomised assignment, and c) where the researchers are involved in the training. In this line, several interventions and trainings are excluded from scientific analyses and thus, it remains difficult to provide consistent evidence of the impact of DPT for the development of socio-emotional competencies. The study of Goldstein and Lerner (2018) conducted on 97 pre-school children, aimed to provide evidence on this and thus controlled the variables shown before, seeking the impact of a pretend play group (PPG) on ToM, altruism, distress towards others and helping behavior, emotion matching and social behavior. The results showed that children in PPG group had lower levels of neutral social behaviors and higher levels of positive social behaviors than block play or reading groups. They also concluded that as children had lower levels of emotional control than the average, the pretend play training did not help with ToM and compassion, but it did help them with emotional regulation and social behaviors.

The importance of developing socio-emotional competencies through drama can be found in the origins of the emotional intelligence concept and in the fundamental role that socio-emotional competencies play in nowadays’ education. Today, there are still several approaches when defining socio-emotional competencies, although they are all coming from the concept of emotional intelligence (EI; Salovey & Mayer, 1990; Mayer & Salovey, 1997). According to Mikolajczak, Quoidbach, Kotsou, and Nelis (2014), the definition of EI still presents some issues within the scientific community, as there is still no consensus among the researchers to identify what means to be *emotionally intelligent*. Consequently, they proposed to gather these capacities under the concept of emotional competencies instead of talking about an intelligence (emotional intelligence). In this line, they defined emotional competencies as “the practical capacity of identifying, understanding, expressing, regulating and using personal and other’s emotions”^i^ (Mikolajczak et al., 2014, p. 3) according to a particular context.

Thus, regarding children’s development and its implication in educational settings, emotional competencies would be the skills that children should have in order to identify, understand, express, regulate and use their own emotions (*intrapersonal level*) and those of their classmates, teachers, friends and people who they interact with (*interpersonal level*), within the school setting. According to Mikolajczak et al. (2014), the interpersonal variant of the “understanding” level of emotional competencies, can be described as the practical capacity of understanding the causes and consequences of others’ perceived emotions. In this line, this capacity would be directly related to the ability of understanding and reacting, in a cognitive, social or affective level, the emotion expressed by another, which is known in academic and popular literature as empathy. According to Davis (1980, 1983) empathy is a complex construct that may be divided into (a) emotional empathy, and (b) cognitive empathy. The emotional branch or side, is related to the emotional process and response of an individual facing another’s emotional reaction (e.g. Eisenberg & Miller, 1987), while the cognitive branch answers to the ability of understanding others’ mental states (e.g. Baron-Cohen & Wheelwright, 2004). The former, is considered by some authors as emotional empathy or just empathy (de Vignemont & Singer, 2006), while the latter, considered as a social cognitive ability to adscribe mental states, such as thoughts, beliefs and emotions to another person is known in literature as ToM (Premack & Woodruff, 1978).

In this line, the development of socio-cognitive skills, such as ToM, and socio-emotional competencies, is intrinsically related to social functioning behaviors and competencies, such as teamwork/collaboration. The latter is considered to be part of the main 21st century skills to develop in education (e.g. Trilling & Fadel, 2009) altogether with creativity and other socio-emotional competencies. In fact, already in 1996, Garaigordobil, Maganto, and Etxeberría (1996) presented evidence on how the experiences lived by children at school had an important impact on children’s prosocial behavior. Social competencies, are detrimental for children development and school is an important modulator of them. According to Burrus and Brenneman (2016), most teachers agree that teamwork/collaboration is a fundamental skill to be learned in the classroom; nevertheless, they are not always sure how to define it, and thus, it gets difficult to find useful tools in order to develop it.

In summary, DPT in schools is an active pedagogy focused on the social and emotional world of children. Studies have presented evidence of the efficacy of DPT related tools and elements in the development of socio-emotional competencies such as ToM and collaborative behavior in elementary school children, although the presented literature regarding the different studies analyzed, concerning drama-based trainings and their impact on socio-cognitive and socio-emotional competencies, do not always present complete reporting, didn’t provide a substantial randomised control, and the only one that did, was focused on preschool children (Goldstein & Lerner, 2018). Teachers agree with the fact of the importance to develop socio-emotional competencies and a collaborative behavior in children, although they are not wide aware of the kind of training that could help them reach this objective. Thus, the main objective of the current study is to conduct a randomised pre-post test study with matching control group in order to present evidence of the efficacy of DPT in promoting socio-emotional competencies, such as ToM skills and a collaborative behavior in elementary school children.

## Method

### Design of Study

We conducted a randomised study including pretest - intervention - posttest design with a matching active control group.

### Participants

French educational system provides mandatory education for all children (foreign and French) from age 3 to age 16. Elementary school comprises 5 levels going from 1st grade (CP) to 5th grade (CM2). Within the French educational system exists two main types of schools: private and public. Within public schools, there is a special type of schools called priority network schools (REP), which are public schools that belong to neighbourhoods with particular social needs. REP schools belong to neighbourhoods with a higher foreign population.

The sample consisted of 126 children: 55 were in 4th grade (CM1; *M* = 9.9, *SD* = 0.35) and 71 in 5th grade (CM2; *M* = 10.8, *SD* = 0.38); 48 assisted a private school (PR), 43 a public school (PU) and 35 a priority school (REP). Children were randomly assigned either to an experimental group participating in a drama pedagogy training (DPT, *n* = 61, *M* = 10.3, *SD* = 0.56) or an active control group participating in Collective Sportive Games (CSG, *n* = 65, *M* = 10.4, *SD* = 0.56). For final analysis, three children were excluded because they were not available for posttests evaluation.

### Materials

#### Independent Variables

##### Trainings

DPT was created by selecting and adapting some drama pedagogy training activities proposed in the spanish program “Programa Juego” (Garaigordobil, 2003) considering that all the activities on the program were already validated. Some other drama pedagogy training activities were taken from the books of García-Huidobro (2004) and Boal (1989). The training consisted of 6 sessions, adjusted to French school time in order to avoid holidays that could interfere with the sessions. At the beginning of each session there was a transition/warming up activity where children expressed how they felt, leaving the standard activities of school and getting prepared for the training. Then, there was the main drama pedagogy training activity(ies), and finally a feedback time in where children expressed their opinions and feelings about the session. Each of the six sessions lasted between 60 to 70 minutes. The differences in the time of each session was due to respect their feedback time. Sessions 2 and 6 had a collective activity, same for both groups. In order to have a clearer idea of the activities within DPT, we will explain a game played in session three: Here, children were asked to create and play a scene with their faces and bodies while the other half of the class had to guess what was going on in the scene, giving details and explanations of their guessing (why they think this or that was going on in the scene). The idea is for children to be able to express something without using words.

CSG was created by selecting popular collective games, taken from Boulo and Olivier (2005) collective games and Orlick’s (1986) cooperative games and adapting this last for French children. It consisted of 6 sessions, adjusted to French school time in order to avoid holidays that could interfere with the sessions. Each of the six sessions lasted 60-70 minutes. Sessions 2 and 6 had a collective activity, same for both groups. As an example of a game played in session three for this group, we will explain the collective game called messengers and combatants: Here, children are divided into two groups, one half has to deliver a message (scarf) and combatants have to avoid the message to be delivered by stealing the message. The idea is for children to work together into a strategy to arrive at the other side of the room and get the message delivered.

#### Dependent Variables

##### Theory of Mind

We developed a French version of the Reading the Mind in the Eyes Test, Child Version (RMET-G; Baron-Cohen, Jolliffe, Mortimore, & Robertson, 1997, Baron-Cohen, Wheelwright, Hill, Raste, & Plumb, 2001). Accordingly to the original version, in this task, children are presented a series of 28 pictures of a pair of eyes from different people expressing an emotion or a mental state. We projected this images on the board. Children were asked to look at the picture in each item and to circle the emotion or mental state that better described the look of the person, choosing it among four words displayed. Three of these words were distractor words while only one was the correct word that matched the emotion or mental state of the person in the picture. The task was scored by adding up the number of items correctly answered. For each image they had around 15 seconds, so between 7 to 10 minutes in total. Reliability score for our sample was acceptable considering our design (α = .64) although a higher reliability score is preferable.

##### Collaborative behaviour

In order to measure collaborative behavior, we used the prisoner’s dilemma (PD) task from of Garaigordobil (1995). For PD, each child has to be sit in two rows back to back with another. They are given two different cards, a red one and a blue one to answer the dilemma. Each couple of children are partners in a robbery and have to decide if betray his/her partner to save him/herself or not. The objective of the game is to stay in prison the less time that is possible. The police gives them a treat. They have one minute to decide, and then pick the decision by raising the corresponding card. They can’t look or talk to their classmates or they are out of the game and lose. Decisions are marked down. The results are said out loud. They cannot comment, and that they are making their decision again, and they can change their decision if they like. Say out loud the results. Explain again: “now you have 30 second to decide if you keep the same answer or if you change your answer. 30 seconds starts now… time is over raise you answer” Write down their answers and say it out loud.

### Procedure

#### Preparation of the Intervention and Cover Story

Authorization letters were sent to parents in order to have their approval for their child participation in the study. In this letter parents were informed of the study, but partially informed of the purpose of it. They were told that children would participate in one of two workshops that might enhance academic achievement through the development of soft-skills, without revealing the expected differences in outcomes for each group/workshop. Teachers were informed about the real purposes of the study, as school directors demanded it in order to agree to participate. They were asked not to tell children about the real purposes of the study in order to avoid emotional predisposition. As a cover story, children were told they would all participate in different collective workshops, explaining them that they will be randomly assigned to one of the two workshops.

#### Random Assignment

After parent’s agreement, each child was given a code. We randomly assigned the codes into a collective sportive games (CSG) group or a DPT group. The randomisation was made by an online free software.

#### Measuring Times and Characteristics

Children in both groups were asked to answer the same different tasks before and after the trainings. There were two pre-tests: one for each variable (ToM and collaborative behavior); and two post-tests: one for each variable aswell.

A week before starting the trainings (T0), children answered the French version of RMET-G in order to measure ToM. In this task they were asked to look at the pair of eyes in each picture and to circle the word that better matched the emotion or mental state of the look of the person among four words displayed with each picture. The week after the task completion, we started with the trainings. In the 2nd session, they were tested by the prisoner’s collaborative task (T1). In the 6th session children were re-tested by the prisoner’s collaborative task (T2). One week after the trainings were finished, children were re-measured on ToM through the RMET-G (T3).

All classes, whether they participated in experimental or control groups, were conducted in the exact same manner: Each class was randomized in order to divide them by group, but times of measurement for ToM were carried on, each time with the whole class. For collaborative behavior, a booklet was followed in order to avoid bias, and thus instructions were given in the exact same way for all classes, groups, and schools.

One researcher of the team conducted both the workshops and assessments following a rigorous booklet in order to avoid bias as much as possible. Other researchers of the team who didn’t have direct contact with children were implicated in data analysis.

## Results

### Preliminary Analyses

Pre-test analyses showed that there were no significant differences in the randomisation, regarding age and sex of the participants, implying an equitative distribution of the groups.

#### Pre-Test Correlational Analysis

In order to see any previous relationship between the expected outcomes, we run a correlational analyses. Results showed no significant correlations.

#### Pre-Test ToM

Analyses also showed no significant differences between the randomised groups in the variable ToM, measured through RMET-G in time T0.

When searching for possible previous ToM differences by class, analyses showed a significant difference on ToM (*t* = -2.87, *p* < .005), with 5th graders scoring higher (*M* = 18.20, *SD* = 3.69) than fourth graders (*M* = 16.24, *SD* = 3.94). These results, are something we expected as ToM is argued to be increased with age (Calero, Salles, Semelman, & Sigman, 2013).

When comparing the effect of the same variable by type of school, results also showed significant differences. ANOVA analyses presented a significant effect of the type of school and a medium effect size on ToM, *F*(2, 123) = 4.17, *p* < .02, η^2^ = 0.06, with the highest mean for the PU school (*M* = 18.6, *SD* = 3.9), then the PR school (*M* = 17.1, *SD* = 3.7), and finally the REP school (*M* = 16.1, *SD* = 3.9).

#### Pre-Test Collaborative Behavior

After the trainings were started, in week 2 (T1), children were measured in collaborative behavior through the prisoner’s dilemma game. Results presented a significant difference between the randomised groups (*t* = 2.79, *p* < .006); with the CSG group (*M* = 3.12, *SD* = 0.13) presenting significant higher mean scores than the DPT group (*M* = 2.62, *SD* = 0.13), after one week of started the trainings. Nevertheless, median and mode scores present no differences between the groups (*Mdn* = 3).

When searching for possible previous Collaborative Behavior differences by class, results showed no significant differences.

When analysing the effect of school on the collaborative behavior variable, measured through prisoner’s dilemma, results showed significant effects, *F*(2, 123) = 7.6, *p* < .001, presenting a medium to large effect size (η^2^ = .11), with PR school scoring higher (*M* = 3.27, *SD* = 0.96) and REP school scoring lower (*M* = 2.43, *SD* = 0.95), both compared to the PU school (*M* = 2.81, *SD* = 1.03).

### Hypothesis Testing

#### Post-Test Correlational Analysis

Pearson’s correlation analyses showed significant weak correlations between ToM and collaborative behavior (measured through PD; *r* = .35; *p* < .001).

#### Post-Test ToM

**Figure 1:**
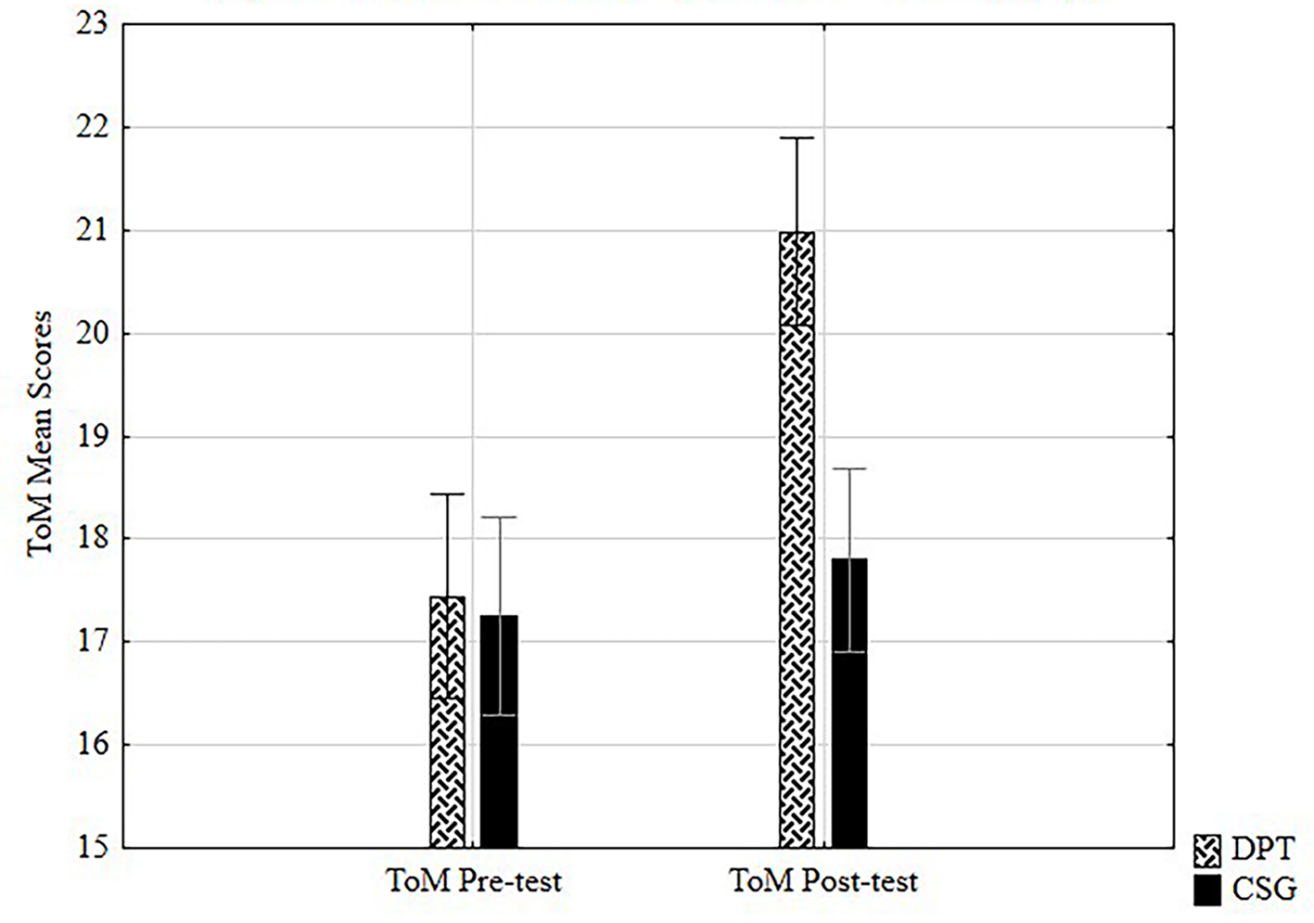
Differences on ToM pre-post test mean scores by group.

As plotted in Figure 1, an ANOVA 2x2 model was run to see the effect of the type of training (DPT, CSG) on the variable ToM regarding the two times of measurement (T0, T3). Results showed significant large effects, *F*(1, 124) = 28.79, *p* < .001, η^2^ = .19, with more important increases on DPT.

#### Post-Test Collaborative Behavior

**Figure 2:**
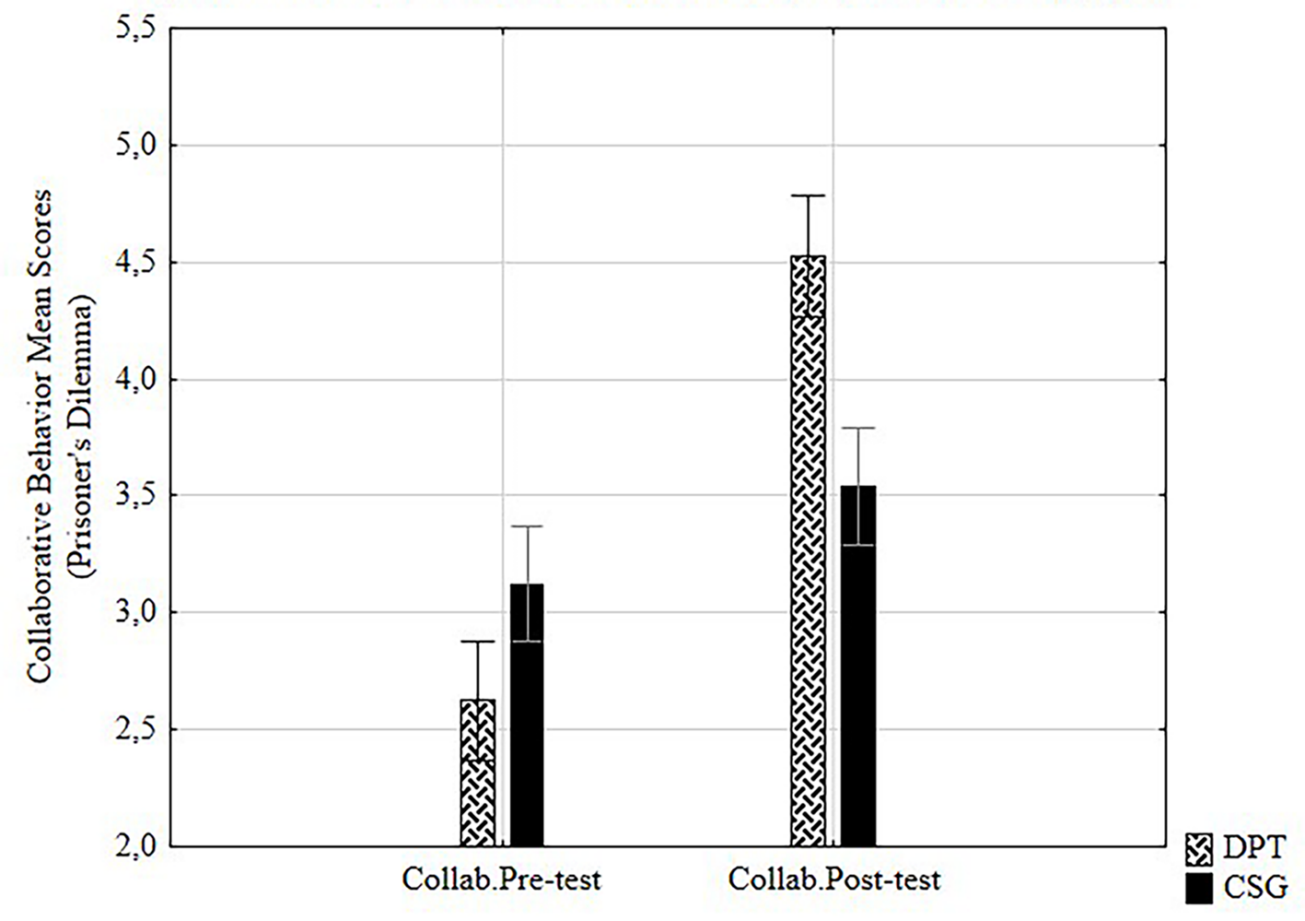
Differences on collaborative behavior pre-post test mean scores by group.

As Figure 2 plots, a two way repeated measures ANOVA analysis was conducted in order to compare the effect of the training (CSG, DPT) on the variable collaborative behavior measured through prisoner’s dilemma, considering the two times of measurement (pre-test and post-test). Results showed significant differences as well as large effect sizes, *F*(1, 124) = 74.83, *p* < .001, η^2^ = .38, higher for DPT.

Further post-test analyses were carried on, comparing means on both ToM and collaborative behavior, in order to confirm the 2x2 ANOVA results.

One way ANOVA for post-test scores showed significant large effects of the training on ToM, *F*(1, 124) = 24.36, *p* < .001, η^2^ = .16, and on collaborative behavior, *F*(1, 124) = 29.8, *p* < .001, η^2^ = .19. Moreover, *t*-test analyses showed significant results in ToM (*t* = -4.94, *p* < .001) with higher mean scores for DPT (*M* = 20.98) than CSG (*M* = 17.80), and in collaborative behavior (*t* = -5.46, *p* < .001) measured through prisoner’s dilemma (PD), also with higher scores for DPT (*M* = 4.52) than CSG (*M* = 3.54).

#### Interaction Effects

##### Type of training by class

**Figure 3:**
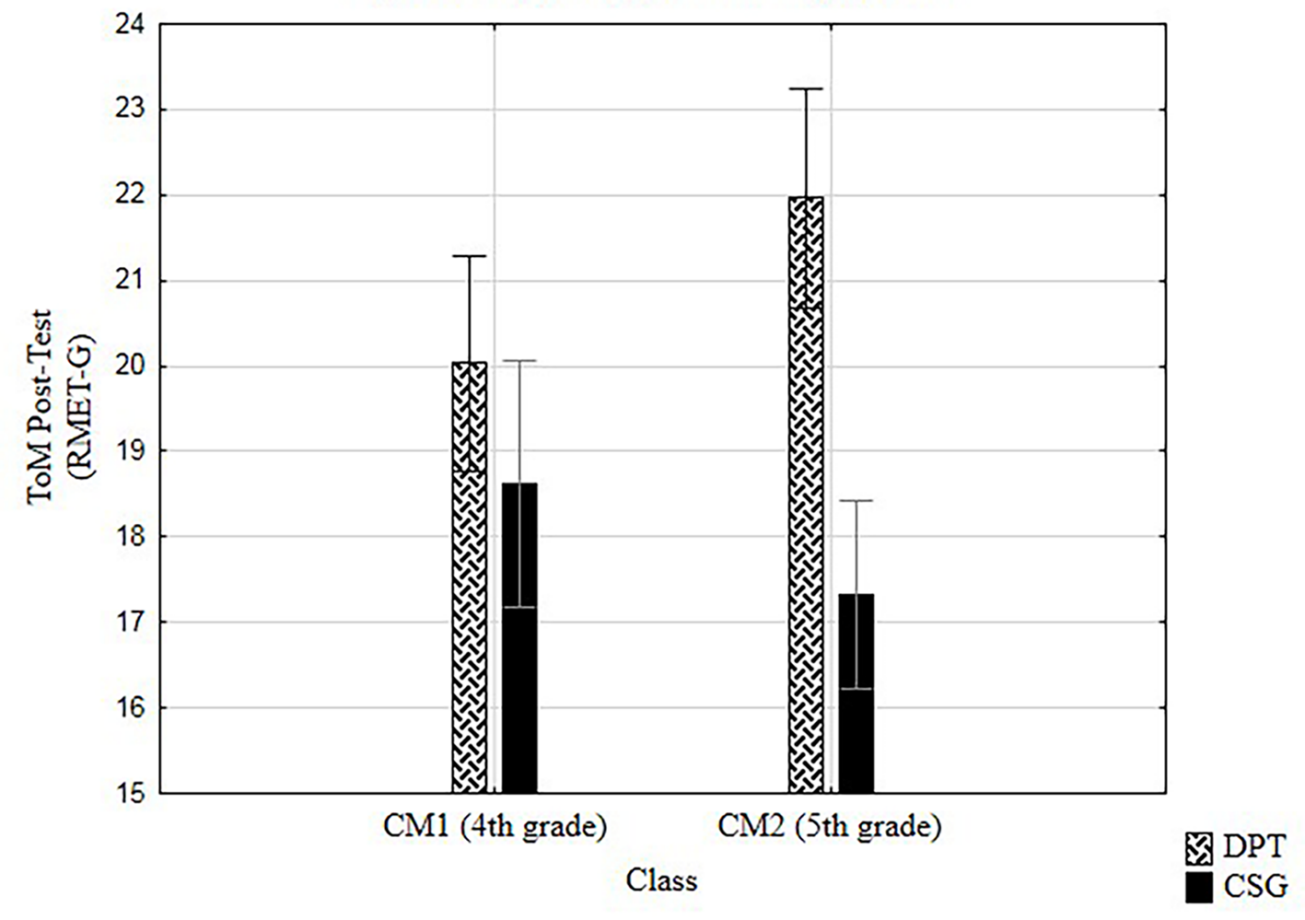
Effects of type of training by class on ToM.

As shown in Figure 3, ANOVA analysis presented a significant effect of the type of training on class, with a small to medium effect size on variable ToM, *F*(1, 122) = 6.32, *p* < .013, η^2^ = .05, with higher means for DPT in both classes.

No significant effect and a small effect size on collaborative behavior-PD (η^2^ = .03) was found by class.

##### Type of training by school

**Figure 4:**
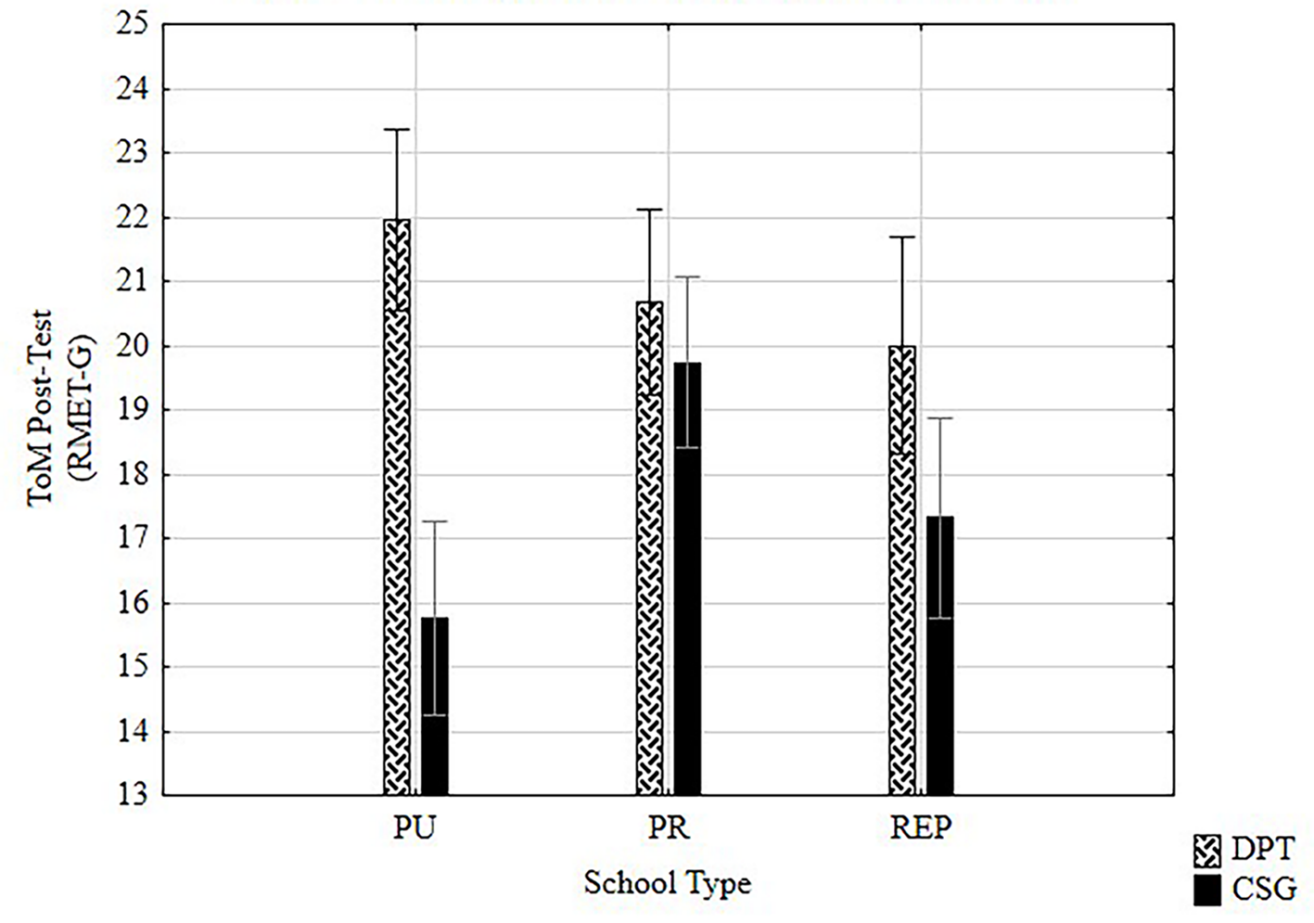
Effect of type of training by type of school on ToM post-test scores.

Figure 4 plots the significant effect and large effect size of the type of training on the type of school on ToM post-test scores, *F*(2, 120) = 6.83, *p* < .002, η^2^ = .102, with higher mean scores for DPT on the three schools.

Further analyses showed no effect of training by type of school on collaborative behavior PD post test scores, nevertheless small effect size was found, presenting scores of η^2^ = .030 with higher means for DPT for three schools.

##### DPT training on ToM by school

Considering the significant effects of the type of training on ToM scores by school type, further ANOVA analyses were made. Looking for interaction between the times of measurement and the type of school in variable ToM, results showed a significant interaction effect, *F*(2, 58) = 5.28, *p* < .008, with large effect size (η^2^ = .15). Highest differences in pre-post scores were found for public (*M* = 18.54, *SD* = 3.9) and REP (*M* = 16.11, *SD* = 3.9) schools.

##### DPT training on ToM and collaborative behavior by class

Analysis showed no significant effect of DPT training on ToM by class, reporting a small effect size (η^2^ = 0.02). No significant effect of DPT training on collaborative behavior by class was found, no effect sizes were reported.

## Discussion and Conclusion

Studies have shown that socio-emotional learning competencies can be taught and learnt at school (Durlak et al., 2011; Ebert, Hoffmann, Ivcevic, Phan, & Brackett, 2015) and at the same time, some researchers insist on the importance to train children as well as teachers on socio-emotional learning competencies, so the benefits can be positive in a personal way but also in a collective way (Fernández-Berrocal, Cabello, & Gutiérrez-Cobo, 2017). More specifically, some authors sustain that ToM and prosocial behavior are two main domains in the field of socio-emotional learning that are important to be developed among children and youngsters (Beauchamp & Anderson, 2010; Rose-Krasnor, 1997). As a recent meta-analysis showed, dramatic play seems to have a positive impact on both of these competencies, even though no rigorous studies have been conducted yet in order to corroborate these effects (Lillard et al., 2013). In the present study, we carried out a randomised protocol in which specific measures for both ToM and collaborative behavior were applied.

ToM can be defined as the ability to read and understand another's mental states through body, face and vocal expression as well as previous knowledge of the other (Goldstein, 2009). In our study, this ability was measured through the RMET-G in which children had to infer mental states through the observation of eyes zones pictures.

Results before the intervention, showed significant differences among schools for ToM for the general sample, implying that depending on the school the children come from, their competencies in this ability were already different regardless the experimental group they belonged to (DPT or CSG). When comparing means, the greater differences were found between the public school and the priority school, with higher scores for the public school. This results could be in part explained by the socio-cultural background of children. In this line, the public school, located in the center of Paris, receives mostly French children with parents that were born in France, and from middle and upper-middle class families, while the priority school, located outside downtown, receives children from different nationalities and/or with parents that were born outside France, some of them from a lower socioeconomic level. Consequently, even if some specific vocabulary is taught at school, an important number of language is learnt at home through parent-child interaction (Hart & Risley, 1992), thus, foreign-parent(s) children would have more difficulties in “foreign” vocabulary acquisition (Mingat, 1991). A more homogeneous French socio-cultural background was present in the public school, with a more foreign socio-cultural background one in the priority school, thus, suggesting that public school children have a more direct access to French vocabulary because they speak the same language at home in contrast to priority school children whose parents (mother or/and father) do not speak French as a first language. We also support this idea based on the fact that private school, where children’s socio-cultural background was very heterogeneous, did not exhibit significant differences with neither of the two other schools.

It was also found a significant difference between both grades’ scores being higher for 5th grade than 4th grade. Nevertheless, this was kind of expected, and can be simply explained by the fact that as children grow up, they acquire more language skills, thus having 5th grade children a larger vocabulary than 4th graders.

Post-test analyses presented encouraging results regarding our hypotheses.

First, correlation analyses showed that there was a significant but weak correlation between ToM and collaborative behavior. This results suggest that it seems to be an important relationship between ToM and collaborative behavior competencies in children that occurs inside a DPT setting. Nevertheless, this relationship is not strong enough to ensure a clear correspondence of ToM with collaborative behavior, thus suggesting more investigation and precisions in this area that should be considered for further research addressing the links between the social competencies that might be enhanced in DPT.

Further on, when comparing the type of training, in order to see the impact of DPT on ToM, both ANOVA analyses and *t*-tests showed effects and significant differences of the type of training on ToM. These significant differences, were statistically higher for DPT group, suggesting that DPT enhanced ToM in both 4th and 5th grade children from all three schools. These results are consistent with previous studies that established a relationship between drama-related games and the development of ToM skills (e.g. Goldstein & Winner, 2012) even though, their results were achieved on a high-school sample. Type of training also showed an effect between pre-test and post-test, confirming post-test analyses. DPT group showed higher differences in scores than CSG, the latter presented a difference of pre-test and post-test scores that was almost invariable. These results are inside the scope of what we predicted, as children who participated in the DPT were trained in emotion and mental states identification through the different DPT methods and activities played. Sessions 1, 3, 4 and 5 were dedicated to emotional identification, expression and communication through play and mini-pretend-plays in where children had to choose an emotion or emotional state and to represent it in an invented collective scene. In this way, children were “obliged” to identify the different characteristics of emotions and the mental states they were to represent, and so learning was acquired through playing, using their minds and bodies to recreate, permitting embodiment, which helped with the understanding of the emotions and mental states. This idea, which defends the theory that embodiment is a direct contributor of the development of the cognitive process of empathy, in which ToM is a fundamental part, is supported by the work of several authors (e.g. Gieser, 2008; Sofia, 2016). Moreover, for some authors, mind cannot be separated from body, as the body is evidence of the mind (Bateson, 2000; Butterworth, 1994). In this means, body and mind cannot be divided because the body is the support of mind expressions.

Regarding the class level, the highest difference in ToM post-test is presented in 5th grade. While DPT group in 4th grade is slightly higher than CSG in 4th grade, scores get clearly higher for DPT in 5th grade. These results suggest that DPT would work better for 5th graders. In both cases, these results only reflect our specific sample and results should be taken carefully as they do not represent the whole French population. Besides, the fact that DPT worked better in 5th grade for developing ToM can be only due to the fact that understanding mental functioning and ascribing mental states to others is in part, a cognitive ability, thus, it should naturally evolve along with children’s cognitive development. In this means, 10-year-old children, that were already turning 11 by the time of the post-tests, have these cognitive abilities more developed. In fact, the theory of stages (Piaget, 1929) describes an evolutionary process of the intellectual capacities which establishes a transformation from concrete operational stage to formal operational stage at the age of 11. Moreover, regarding ToM, Perner and Wimmer (1985), confirm a natural evolution in the attribution of second-order beliefs in their work with children aged 5 to 10-year-olds.

Regarding the difference between pre-test and post-test repeated measures ANOVA found that DPT on ToM task had differences considering the type of school. As shown in results, PU school showed almost no increase between pre and post ToM tasks in comparison with REP and PR schools which post scores were between 2 and 3 points of difference.

On the other hand, collaborative behavior may benefit children’s social relationships (Schellenberg, Corrigall, Dys, & Malti, 2015) as is encompassed in prosocial behavior which appears to be one of the key elements that benefits social interaction (Eisenberg, Spinrad, & Knafo-Noam, 2015). Moreover, in 2006, Barry & Wentzel (2006) established that collaborative behaviors develop a sense of security among friends and peers. In our study, we analysed collaborative behavior from two perspectives; an objective one through task performance and a more subjective one, through the observation of children’s behaviors.

The state of the art showed that there were already differences among children from different schools, being always PR school children who had the more higher scores in collaborative behavior. After questioning teachers and directors from the three schools involved, there’s still no clear answer that could explain this higher collaborative behavior in comparison to the other schools. No specific program was held before our intervention, and all three schools offered the same kind of extracurricular activities.

Girls showed to be more collaborative than boys. Observations in both pre-test and post-test showed that they tended to be more encouraging than boys in their groups, but also dynamics observed among groups constituted mostly of girls presented less anti-collaborative behaviors and showed a more federated way of work. This is consistent with Garaigordobil (2009) findings who established that girls are more collaborative than boys.

Even though group randomisation was made completely blinded and through an online free software, *t*-test results showed a significant difference between intervention groups. This means that there were already differences in collaborative behavior scores between the experimental (DPT) and the active control group (CSG). CSG started the interventions presenting an advantage over the DPT group. When teachers were asked about differences they could perceive between both groups of their class after they were constituted, some of them agreed that DPT group was composed by “more difficult students than the other group” and that they felt the difference of working in the class with the other half (CSG) while the DPT session was being carried out. They reported they were able to “work better without that part of the class”. Nevertheless, this wouldn’t be sufficient to explain these differences, so we rely on the fact that the evaluation of collaborative behavior was carried on one week after the groups were constituted. With this timing in mind, we might infer that after a week of intervention, children already had created some particular relationships, group identification or bonding links with their classmates in their corresponding groups (DPT and CSG), which would had interfered on the second week, when the evaluation of collaborative behavior was carried on.

Finally, when testing our hypothesis, for collaborative behavior we found significant differences between the two groups, being higher for DPT. Both *t*-test analysis and ANOVA confirmed the hypothesis. This means that children in the DPT group increased their collaborative behavior during the intervention showing important differences when compared to their CSG classmates. PD task measure how the children respond and solve a situation in which they have to decide whether to collaborate to their partner or no. Children are offered the possibility to escape from prison, even though this means that their partner will stay in prison for some years. At the beginning, DPT group presented less collaboration, showing answers that in great majority chose not to collaborate and so escape from prison. After DPT was conducted, children appeared to develop more consciousness on the need to collaborate with others, and they decided to collaborate in the PD task, staying a few years in prison in order to not let their partner alone in prison for even more years. This behavioral change confirms that a DPT enhances collaborative behavior, and that a training based on expressive art such as drama can facilitate some socio-emotional learning competencies. Regarding this, the International School for Interdisciplinary Studies, in Canada establishes that expressive arts might improve social learning by giving children the opportunity to work in group, collaborate, and create a social support network (ISIS, 2004). Our results are consistent with several studies that confirm the positive effect of drama and drama based trainings over collaborative behavior and social skills development (e.g. Garaigordobil, 2003; Joronen, Konu, Rankin, & Astedt-Kurki, 2012; Wright, John, Ellenbogen, Offord, Duku, & Rowe, 2006). Nevertheless, they are not consistent with Lee et al. (2015) who explained that even though drama-based pedagogies have been supported as being an effective way to foster prosocial behaviors studies analysed in their meta-analyses were not consistent.

### Limitations and Future Perspectives

One of the main concerns we find in this study is related to the way instruments were used. The fact that the pre- and post-tests were identical could imply that there were learning effects on children for the post-tests and that increased scores were in part due to this fact. Nevertheless, we consider that if this was the case, this learning effect would have affected both groups equally as both groups answered the exact same tasks at the same time, so it shouldn’t have a direct impact on the significant differences between the groups. Another limitation we appreciate is that the RMET-G task only measures one dimension of ToM, and moreover, its reliability can be discussed. Unfortunately there are not many tasks for measuring ToM in the range of age we worked with, as most reliable tasks are constructed for younger children and only few are translated and validated in France. It could also be argued that as DPT is a methodology focused on the work with the internal world of participants it might have a direct impact in socio-cognitive tasks such as the RMET-G. We propose, for future studies, an intervention considering different forms of the same task in order to discard the issue of learning effect, and the construction of a different scale that can measure better what was intended in the beginning of the study, or even the use of additional tests for measuring ToM in order to address the issue of reliability and measured dimensions of ToM. This also opens a possibility for further research considering the different approaches to emotional vocabulary and content that might be involved depending on the different socio-cultural backgrounds. On the other hand, the significant differences found in the pre-test scores for collaborative behavior should also be considered carefully. We propose a future intervention that considers carrying on all variable testing tasks before any session had already started in order to avoid the bias of bonding in the different groups.

Another limitation we observed is related to the analyses chosen. Considering that differences among children were significant among the type of schools, it could have been interesting to run further analyses in order to control results regarding this issue. A future study should consider a multi-level analysis.

Finally, no follow-up study was conducted in order to measure both of the developed competencies. In this line, we propose to conduct a new study, probably using a longer intervention of DPT in order to see the possible follow-up impact on ToM and collaborative behavior scores, and why not, on other SEL competencies, as previous studies suggest.

In summary, DPT showed to be an effective method to develop ToM and collaborative behavior in 9 to 10-year-old children engaged in three different French school contexts, although further work is needed in order to see the long term impact of this pedagogy.
